# ﻿The *Ganodermacurtisii* lineage (*Basidiomycota*, *Polyporaceae*) in the Neotropics: *Ganodermamexicurtisii* sp. nov. from pine-oak forests in Mexico

**DOI:** 10.3897/imafungus.16.154828

**Published:** 2025-07-04

**Authors:** Milay Cabarroi-Hernández, Cony Decock, Laura Guzmán-Dávalos, Mabel Gisela Torres-Torres, Gerardo Lucio Robledo, Alma Rosa Villalobos-Arámbula, Zurizadai Martínez-Velázquez, Virginia Ramírez-Cruz, Mario Amalfi

**Affiliations:** 1 Departamento de Botánica y Zoología, Universidad de Guadalajara, Apdo. postal 1–139, Zapopan, CP 45147, Jalisco, Mexico; 2 Mycothèque de l’Université Catholique de Louvain, Croix du Sud 2 box L7.05.06, B-1348, Louvain-la-Neuve, Belgium; 3 Universidad Tecnológica del Chocó, Ciudadela Medrano, Quibdó, Chocó, Colombia; 4 Universidad Nacional de Córdoba, Facultad de Ciencias Agropecuarias, Centro de Transferencia de Bioinsumos, CP 5000, Ciudad Universitaria, Córdoba, Argentina; 5 Consejo Nacional de Investigaciones Científicas y Técnicas, Córdoba, Argentina; 6 Departamento de Biología Celular y Molecular, Universidad de Guadalajara, Zapopan, Jalisco, Mexico; 7 Secretaría de Ciencias, Humanidades, Tecnología e Innovación, Mexico City, Mexico; 8 Meise Botanic Garden, Nieuwelaan 38, 1860 Meise, Belgium; 9 Fédération Wallonie–Bruxelles, Service Général de l’Enseignement Supérieur et de la Recherche Scientifique, 1080 Bruxelles, Belgium

**Keywords:** Molecular systematics, Neotropical polypores, *Polyporaceae*, species complex, taxonomy

## Abstract

*Ganodermacurtisii*, a potential medicinal species due to the presence of various lucidenic acids, was originally described from the southeastern United States. Controversy subsequently developed as it became clear that this was not a single species but a complex. In the present study, 39 collections from the *G.curtisii* complex, including 30 collections originating from four different states of Mexico and type specimens were analyzed from a phylogenetic, morphological, and ecological point of view. The phylogenetic relationships within the *G.curtisii* complex were analyzed using sequence data from ITS, *tef1*, *rpb1*, and *rpb2* regions. A total of six clades were resolved within the *G.curtisii* complex, that corresponds to *G.curtisii*, *G.myanmarense*, *G.ravenelii*, *G.sichuanense*, *Ganoderma* sp. from Costa Rica, and a clade comprising several collections previously named *G.curtisii* from Mexico, described here as *Ganodermamexicurtisii***sp. nov.** Furthermore, *G.meredithiae* is confirmed as a synonym of *G.curtisii*.

## ﻿Introduction

*Ganoderma* P. Karst. (*Basidiomycota*, *Polyporaceae*) is a highly diverse genus, whose taxonomy and phylogeny are still in a state of flux as emphasized recently by comprehensive phylogenetic studies that have revealed numerous lineages or species complexes (e.g., Costa-Rezende et al. 2020; He et al. 2022, 2024). For instance, Cabarroi-Hernández et al. (2019) revised the so-called *G.resinaceum* / *weberianum* lineage and confirmed that *G.weberianum* sensu Steyaert (1980), previously considered a widely distributed species, represented a species complex, with at least two Neotropical endemics, *G.mexicanum* Pat. and *G.parvulum* Murrill, with a third clade tentatively attributed to *G.perzonatum* Murrill, and a biogeographical dichotomy between the Paleotropics versus Neotropics.

*Ganodermacurtisii* (Berk.) Murrill is another species complex with a presumed wide distribution. Most records of *G.curtisii* originate from the southeastern United States down to Mexico (e.g., Murrill 1908; Steyaert 1980; Torres-Torres et al. 2005; López-Peña et al. 2016; Loyd et al. 2018a, 2019; Mendoza-Churape et al. 2024). Loyd et al. (2018a) restricted its distribution to North America. However, the species was also reported from Brazil (Torres-Tores et al. 2012), Costa Rica (Mardones et al. 2023), and Cuba (Cabarroi-Hernández et al. 2023). Out of the Neotropics, it was mentioned in Africa (Lloyd 1915), China (Zhao 1989), and India (Bhosle et al. 2010).

*Ganodermacurtisii* was first described as *Polyporuscurtisii* Berk., from South Carolina, USA (Berkeley and Curtis 1849). Six specimens were mentioned in the protologue, Curtis 549, 577, 908, 979, 1131, 1525 (K), without designation of a type, which was the common way of proceeding then. Murrill (1908) transferred the species to *Ganoderma* and cited some exsiccatae, including “Rav. Fungi Am. 5, 47 (corrected as 471, Murrill 1908), Rab.-Wint. Fungi Eur. 3430, and Ellis, N. Am. Fungi 802”, but none of those listed in the protologue. Murrill (1908) reported the species from New York to Florida, and west to Texas, growing on deciduous trees such as oaks, *Eucalyptus*, maples, and others.

Steyaert (1980) noted that the original specimens of Curtis, for a time preserved at K, were “no longer extant” and suggested that a neotype be designated. Previously, Ravenel had labeled the specimen Ravenel 2936 (USA, South Carolina, K) as neotype; however, this neotypification was not officially published. Steyaert (1980) studied Ravenel 2936 and noted that it contained two basidiomes, in different conservation states. After a careful comparison of both, he concluded that they represented two morphospecies differentiated by the combination of the presence of melanoid substances deposits in the context and broadly ellipsoid to ovoid basidiospores vs. absence of melanoid substances and long ellipsoid basidiospores.

Steyaert (1980) considered the first morphospecies as corresponding to *P.curtisii*. Berkeley and Curtis (1849) had already mentioned in the protologue of *P.curtisii* a “substance [in the context] …. traversed by laccate lines parallel to the surface” that could be equated to deposits of melanoid substances in the context. However, due to its bad conservation state, Steyaert (1980) concluded that this basidiome would not be suitable for typification and then designated the specimen Coker s.n., XI–1923, North Carolina, USA (RLS.63.K.59 & K.58) (K, fragm. in BR), as neotype. At the same time, he described the second morphospecies based on the specimen Ravenel 2936 (RLS. 55. K.1) as *G.ravenelii*Steyaert (1980). Later on, Adaskaveg and Gilbertson (1988) segregated *G.meredithiae* Adask. & Gilb. from *G.curtisii* based on substrate affinities (*Pinus* vs. *Quercus*) and morphological and cultural features, emphasizing “the frequently lobed or branched pilocystidia” and a slower growth rate *in vitro* on MEA. However, multilocus phylogenetic inference (ITS, *tef1*, *rpb1*, and *rpb2*) confirmed that *G.meredithiae* grouped within the *G.curtisii* clade, which led to the conclusion that both names are synonymous (Loyd et al. 2018a). Nonetheless, given its host preference, Loyd et al. (2018a) considered the specimens on coniferous as G.curtisiif.sp.meredithiae.

Loyd et al. (2018a) showed also that the North American *G.curtisii* and *G.ravenelii*, together with the Asian *G.sichuanense* J.D. Zhao & X.Q. Zhang (Zhao et al. 1983) (= *G.lingzhi* Sheng H. Wu, Y. Cao & Y.C. Dai [Cao et al. 2012]) formed the “curtisii clade”, with *G.curtisii* and *G.ravenelii* on the one hand, and *G.sichuanense*, on the other, having disjoint distributions in North to Central America and East Asia, respectively (Loyd et al. 2018a).

From a medicinal perspective, *G.curtisii* stands out for its abundance of lucidenic acids (Welti et al. 2015). This class of triterpenoids is the second most represented in different *Ganoderma* species (e.g., Weng et al. 2010), and has demonstrated cytotoxicity against various cancer cell lines, anti-inflammatory and antioxidant properties, and hepatoprotective effects, as well as antiviral activity, and might play a role in regulating blood sugar levels, suggesting potential benefits for diabetic patients (e.g., Zhu et al. 1999; Weng et al. 2008; Sato et al. 2009). Specifically, in Mexico, Islas-Santillán et al. (2018) documented the presence of sterols with important biological activities in extracts of basidiomata of *G.curtisii* growing in *Pinus-Quercus* forest from the state of Hidalgo.

In Mexico, *G.curtisii* has been reported from many states, however, the name has been applied indistinctly to almost any stipitate and laccate *Ganoderma*, especially those growing in *Quercus-Pinus* forests (e.g., Guzmán 1977; Torres-Torres and Guzmán-Dávalos 2005; Torres-Torres et al. 2015; López-Peña et al. 2016; Cappello-García et al. 2023). Recently, Mendoza-Churape et al. (2024) also reported *G.curtisii* as the cause of white rot root and stem disease in *Perseaamericana* orchards in the State of Michoacán. Nevertheless, Torres-Torres and Guzmán-Dávalos (2005) questioned the wide morphological variability within *G.curtisii* as it was accepted then and consequently the morphological species concept, suggesting that it could encompass several taxa. The present study aims to understand the *G.curtisii* complex in North and Central America and to evaluate the taxonomic status and phylogenetic relationships of specimens of the *G.curtisii* complex from Mexico integrating multilocus DNA sequences analysis, morphological data, and substrate affinity.

## ﻿Methods

### ﻿Studied material

Thirty-nine specimens of *Ganoderma* labeled as *G.curtisii* and *G.ravenelii* kept at BR, ENCB, F, IBUG, and MUCL (herbarium acronyms follow Thiers [2024]), including the isoneotype of *G.curtisii* and the holotype of *G.ravenelii*, were studied. Additionally, 20 specimens were collected by the present authors from central and western Mexico. The type culture of *G.meredithiae* (CBS) was also revised and sequenced.

### ﻿Morphological study

For the microscopical observations, freehand sections were mounted in 5% KOH solution or Melzer’s reagent and observed under optical microscopes (Axioscope 40 Carl Zeiss or Olympus BX–51). Images were captured using the AxioVision 4 software on the Axioscope. Micromorphological features such as basidiospore characteristics and the shape and size of the cuticular cells were analyzed for species diagnosis. Basidiospores, basidia, and pileal elements were randomly selected, the last ones near the pileal center. At least 30 structures of each mature specimen were measured. Basidiospores were measured without considering the apical umbo when not shrunk. The formation of chlamydospores was examined in three strains grown on malt extract agar medium at 25 °C for 4 weeks according to Bazzalo and Wright (1982). In the case of the macromorphological characteristics, the shape and color of the pileus and features of the context, such as color and presence of resinous bands, were considered. Color terms follow Kornerup and Wanscher (1963) and terms in descriptions were defined by Torres-Torres and Guzmán-Dávalos (2012) and Cabarroi-Hernández et al. (2023).

### ﻿DNA extraction, amplification, and sequencing

Genomic DNA was extracted from mycelia cultures, fresh specimens, and herbarium specimens using three protocols: (1) CTAB method with 1% polyvinylpyrrolidone (PVP) (Palomera et al. 2008), (2) salt-extraction method with 1% PVP (Aljanabi and Martinez 1997), and (3) Wizard Genomic DNA Purification Kit (Promega). Four independent loci (ITS, partial *tef1*, partial *rpb1*, and partial *rpb2*) were amplified by PCR. Each 53 μL (ITS and *tef1*), or 55 μL (*rpb1* and *rpb2*) PCR reaction contained 50 μL of PCR mix [35 μL of PCR water, 6 μL of 10× Taq reaction buffer without MgCl_2_, 3 μL of 50 mM MgCl_2_, 3 μL of 5 mM dNTP, 3 μL of 2 μg/μL Bovine Serum Albumine (BSA)], 0.5 μL or 1.5 μL of each 10 μM primer, 0.15 μL of Platinum™ Taq DNA Polymerase High Fidelity (5U/μL), and 2 μL of DNA template (1:5 dilution). Primers G–ITS–F1/ITS4B (Cao et al. 2012), ITS1F/ITS4B (Gardes and Bruns 1993) or ITS1F/ITS4 (White et al. 1990) were used to amplify the rDNA ITS (ITS1, 5.8S, and ITS2). Primers bRPB2–6F/bRPB2–7.1R (Matheny 2005) for *rpb2*, EF983F/EF2218R (Rehner and Buckley 2005; Matheny et al. 2007) for *tef1*, and RPB1–Gano18F and RPB1–Gano958R (Loyd et al. 2018a) for *rpb1* were used to amplify these regions.

PCR amplifications were performed in an ESCO Swift MaxPro thermocycler with a thermal profile as described by Guzmán-Dávalos et al. (2003) for ITS, except that the annealing temperatures [55 °C (ITS1F/ITS4B) and 59 °C (G–ITS–F1/ITS4B)] followed Cao et al. (2012). PCR cycling conditions for *rpb2* and *tef1* were: 95 °C for 5 min, 35 cycles at 95 °C for 1 min, 55 °C (*rpb2*) or 59 °C (*tef1*) for 1 min, 72 °C for 2 min, and a final extension at 72 °C for 10 min. For *rpb1* we performed an initial denaturation at 95 °C for 10 min, followed by 35 cycles at 95 °C for 30 sec, 62 °C for 30 sec, 72 °C for 1 min, and a final extension step of 72 °C for 5 min. PCR products were purified with the GFX^TM^ PCR DNA Purification Kit (GE Healthcare). Purified products (GFX) were sent to the University of Arizona, MUCL, Macrogen Ltd. (Europe), and LaniVeg (CUCBA, University of Guadalajara) for Sanger sequencing. PCR and amplification protocols for the ITS, *rpb1*, *rpb2*, and *tef1* genomic regions from cultures were described in Decock et al. (2007), Amalfi et al. (2010), and Loyd et al. (2018a). Sequences were assembled and edited in Sequencher™ 4.8 software (Gene Codes Corp.).

### ﻿Phylogenetic analyses

A combined data set (ITS, *tef1*, *rpb1*, and *rpb2* genes) comprising sequences from 51 collections (including the outgroup) was constructed and used for further phylogenetic analyses (Table 1). Sequences of *G.lucidum* Murrill were selected as the outgroup following Cabarroi-Hernández et al. (2023). In each data set the sequences were automatically aligned with MUSCLE (Robert 2004) and manually adjusted using PhyDe (Müller et al. 2010). Potentially ambiguously aligned segments were also detected using the Gblocks 0.91b program (Castresana 2000) with the following parameter settings: minimum number of sequences for a conserved position = 25 (minimum possible); minimum number of sequences for a flank position = 25 (minimum possible); maximum number of contiguous non-conserved positions = 4 bp; and minimum block size = 4 bp and gaps allowed within selected blocks in half of the sequences. The assignment of codon positions was confirmed by translating nucleotide sequences into predicted amino acid sequences using MacClade 4.0 (Maddison and Maddison 2005) and then compared with several annotated *Ganoderma* sequences available on GenBank.

**Table 1.** Species, specimens, and DNA sequences of *Ganoderma* used in this study with their geographic origin. Sequences marked in bold were obtained for this work (Hgo = Hidalgo, Jal = Jalisco, Mich = Michoacán).

Species	Voucher/strain	Locality	GenBank accession numbers				Reference
			ITS	*rpb2*	*rpb1*	*tef1*	
*G.curtisii*	CBS 100132	NC, USA	JQ781849	KJ143967	KJ143947	KJ143927	Zhou et al. 2015
	CBS100131	NC, USA	JQ781848	KJ143966	KJ143946	KJ143926	Zhou et al. 2015
	MUCL 47086	Cuba	OQ079172	**PV702323**	**PV711346**	**PV738120**	Cabarroi-Hernández et al. 2023 / **This study**
	MUCL 47088	Cuba	OQ079173	**PV702321**	**PV711347**	**PV738119**	Cabarroi-Hernández et al. 2023 / **This study**
	MUCL 49396	USA	**PV697562**	**PV702322**	**PV711348**	**PV738121**	**This study**
	UMNGA1	GA, USA	MG654117	MG754857	MG754791	MG754731	Loyd et al. 2018a
	102NC	NC, USA	MG654074	MG754851	–	MG754727	Loyd et al. 2018a
	UMNFL28	FL, USA	MG654097	MG754856	MG754788	MG754728	Loyd et al. 2018a
	136FL	FL, USA	MG654148	MG754852	MG754783	–	Unpublished
	AL–M8	GYO Reishi Kit	MH160063	–	–	–	Loyd et al. 2018b
	AL–M17	GYO Reishi Kit	MH160072	–	–	–	Loyd et al. 2018b
	UMNFL60	FL, USA	MG654105	–	MG754789	MG754729	Loyd et al. 2018a
	UMNNC3	NC, USA	MG654130	–	MG754794	MG754732	Unpublished
	UMNSC2	SC, USA	MG654141	–	MG754797	MG754733	Unpublished
	GA–63	Costa Rica	OQ845460	–	–	–	Mardones et al. 2023
	GA–00	Costa Rica	OQ845458	–	–	–	Mardones et al. 2023
	GA–65	Costa Rica	OQ845461	–	–	–	Mardones et al. 2023
	UMSH 18	Mich., Mexico	OR485049	–	–	–	Mendoza-Churape et al. 2023
	UMSH 10	Mich., Mexico	OR485047	–	–	–	Mendoza-Churape et al. 2023
	UMSH 12	Mich., Mexico	OR485048	–	–	–	Mendoza-Churape et al. 2023
*G.lucidum*	MUCL 35119	France	MK554779	MK554752	–	MK554719	Cabarroi-Hernández et al. 2019
	MUCL 31549	France	MK554777	MK554765	–	MK554730	Cabarroi-Hernández et al. 2019
	UMNUT7	UT, USA	MG654071	–	–	MG754726	Loyd et al. 2018a
*G.meredithiae*	CBS 271.88 (holotype)		MH862131	**PV702324**	**PV711349**	**PV738122**	Vu et al. 2018/**This study**
	UMNFL50	FL, USA	MG654103	MG754862	MG754806	MG754735	Loyd et al. 2018a
	UMNFL64	FL, USA	MG654106	–	MG754807	MG754863	Loyd et al. 2018a
	UMNFL80	FL, USA	MG654109	–	MG75479	MG754730	Unpublished
	124FL	FL, USA	MG654188	MG754861	MG754805	MG754734	Loyd et al. 2018a
*G.mexicurtisii*	L. Guzmán-Dávalos 11569 (IBUG), sequence Gp30 (paratype)	Jal, Mexico	**PV714873**	**PV702314**	–	**PV711351**	**This study**
	O. Castro-Jauregui 146 (IBUG), sequence Gp32 (paratype)	Jal, Mexico	**PV714874**	**PV702317**	–	**PV711358**	**This study**
	O. Castro-Jauregui 365 (IBUG), sequence Gp36 (paratype)	Jal, Mexico	**PV714875**	**PV702316**	–	**PV711352**	**This study**
	M.X. Haro-Luna 394 (IBUG), sequence Gp37 (paratype)	Jal, Mexico	**PV714876**	–	–	**PV711355**	**This study**
	L. Guzmán-Dávalos 11735 (IBUG), sequence Gp38 (paratype)	Coah, Mexico	**PV714877**	–	–	**PV711354**	**This study**
	L. Guzmán-Dávalos 11754 (IBUG), sequence Gp39 (paratype)	Jal, Mexico	**PV714878**	**PV702320**	–	**PV711356**	**This study**
	M. Cabarroi 12 (IBUG), sequence Sc389 (paratype)	Jal, Mexico	**PV714879**	**PV702319**	–	**PV711357**	**This study**
	**L. Guzmán-Dávalos 12340 (IBUG), sequence Gp29 (holotype)**	Jal, Mexico	**PV714880**	**PV702318**	–	**PV711353**	**This study**
	C. Decock MUCL 52280 (paratype)	Hgo, Mexico	KF963259	**PV702325**	–	**PV738123**	Welti et al. 2025/**This study**
*G.myanmarense*	MFLU 19–2167 (holotype)	Myanmar	MN396330	–	–	–	Du et al. 2023
	MFLU 19–2211 (paratype)	Myanmar	MN396329	MN423134	–	MN423167	Du et al. 2023
	MQN002	Nepal	AB811848	–	–	–	Unpublished
*G.pulverulentum*	MUCL 38859	Argentina	OQ079177	OQ401033	–	OQ561004	Cabarroi-Hernández et al. 2023
	MVHC 5583/GM21	Uruguay	MN191575	–	–	OQ378923	Morera et al. 2020 / Cabarroi-Hernández et al. 2023
*G.ravenelii*	MS187FL	FL, USA	MG654211	MG754865	MG754813	MG754745	Loyd et al. 2018a
	UMNFL188	FL, USA	–	–	MG754815	MG754746	Loyd et al. 2018a
	151FL	FL, USA	MG654208	–	–	–	Unpublished
*G.sichuanense* (as *G.lingzhi*)	Dai 12479 (IFP)	China	JQ781864	JX029979	–	JX029975	Cao et al. 2012
	Dai 12574 (IFP)	China	KJ143908	JX029981	–	JX029977	Zhou et al. 2015
	Dai 20895	China	MZ354904	MZ245413	–	MZ221668	Sun et al. 2022
	JZB2114009	China	MH294306	MH294406	–	MH294373	Unpublished
	Wu 1006-38 (holotype, *G.lingzhi*)	China	JQ781858	JX029980	JX029984	JX029976	Cao et al. 2012
	CGMCC 5.2175 (epitype, *G.sichuanense*)	China	KC662402	KC662404	–	–	Yao et al. 2013

Phylogenetic analyses were performed separately for each individual and concatenated loci using Bayesian inference (BI) as implemented in MrBayes 3.2.6 (Ronquist et al. 2012) and maximum likelihood (ML) as implemented in RAxML 7.0.4 (Stamatakis et al. 2008). Models of evolution for BI were estimated using the Akaike information criterion (AIC) as implemented jModelTest 2.1.10 (Darriba et al. 2012). The data set was subdivided into eight partitions: (ITS–5.8S) (*rpb2* 1^st^ and 2^nd^ codon positions) (*rpb2* 3^rd^ codon position) (*rpb1* 1^st^ and 2^nd^ codon positions) (*rpb1* codon 3) (*tef1* codons 1 and 2) (*tef1* codon 3) (combined introns of *tef1*, *rpb1*, and *rpb2*). The best-fit models for each partition were implemented as partition-specific models within partitioned mixed-model analyses of the combined data set, and all parameters were unlinked across partitions. Bayesian analyses were implemented with two independent runs, each with four simultaneous independent chains for 6 million generations, starting from random trees and keeping one tree every 1000^th^ generation. All trees sampled after convergence (average standard deviation of split frequencies <0.01) and confirmed using Tracer 1.4 (Rambaut and Drummond 2007; Rambaut et al. 2018) were used to reconstruct a 50% majority rule consensus tree and to calculate Bayesian posterior probabilities (PPs). PP of each node was estimated based on the frequency with which the node was resolved among the trees sampled using the 50% majority rule consensus option (Simmons et al. 2004), where a probability of 0.95 was considered significant. Maximum likelihood (ML) searches were conducted with RAxML involving 1000 replicates under the GTRGAMMAI model, with all model parameters estimated by the program. In addition, 1000 bootstrap (ML BS) replicates were run with the same GTRGAMMAI model. In order to force RAxML software to search for a separate evolution model for each dataset, we provided an additional alignment partition file to the software. Clades with ML BS values of 75% or greater were considered supported by the data. Alignment was submitted to Figshare (10.6084/m9.figshare.29218322).

Nucleotide sequences are phylogenetically informative until they reach the substitution saturation, especially in coding sequences, where saturation will be more pronounced in the rapidly evolving third codon position. At this point, it is no longer possible to deduce whether an observed similarity between a pair of sequences results from their common ancestry or whether this has occurred by chance (Jeffroy et al. 2006). To detect the possible bias from substitution saturation, we tested the first, second, and the third codon position of the coding region studied (*tef1*, *rpb1*, and *rpb2*) as well as the non-coding loci (ITS–5.8S, *tef1*, *rpb1*, and *rpb2* introns) by using Xia’s test (Xia et al. 2003; Xia and Lamey 2009), as implemented in DAMBE (Xia and Xie 2001). To confirm the results of the Xia’s method, we also plotted transitions and transversions at the first, second, and third codon positions against Tamura-Nei genetic distances with the aid of the DAMBE package, with an asymptotic relationship indicating the presence of saturation. Before combining the data partitions, topological incongruence between the datasets was assessed using 1000 replicates of ML BS under the same models described above, on each locus separately. Paired trees were examined for conflicts only involving nodes with ML BS > 75% (Mason-Gamer and Kellogg 1996; Lutzoni et al. 2004; Reeb et al. 2004) and compared with the software compat.py (Kauff and Lutzoni 2002), available at https://www.lutzonilab.net/downloads. A conflict was assumed to be significant when two different relationships for the same set of taxa (one being monophyletic and the other non-monophyletic) were observed in rival trees.

### ﻿Distribution map

A distribution map was generated in ArcMap 10.5 (ESRI 2010) using geographic information of the specimens studied. When the geographic information was lacking, it was inferred using digital cartography following the criteria of Wieczorek et al. (2004).

## ﻿Results

### ﻿Phylogenetic analyses

The combined dataset comprised 138 sequences and 3581 positions including gaps (ITS: 579 characters, *tef1*: 1048, of which 875 in the exon partition and 174 in the combined intron partition; *rpb1*: 1016, of which 540 in the exon partition and 476 in the combined intron partition; *rpb2*: 937, of which 859 in the exon partition and 78 in the combined intron partition). The evolutionary models that best fit the individual datasets according to the AICc criterion were HKY for ITS, the 1^st^, 2^nd^, and 3^rd^ codon positions of *rpb1*, and 1^st^ and 2^nd^ codon positions of *tef1*, K80 for 1^st^ and 2^nd^ codon positions of *rpb2*, GTR for 3^rd^ codon position of *rpb2*, GTR+G for the 3^rd^ codon position of *tef1*, and HKY+G for combined introns partitions.

**Figure 1.**
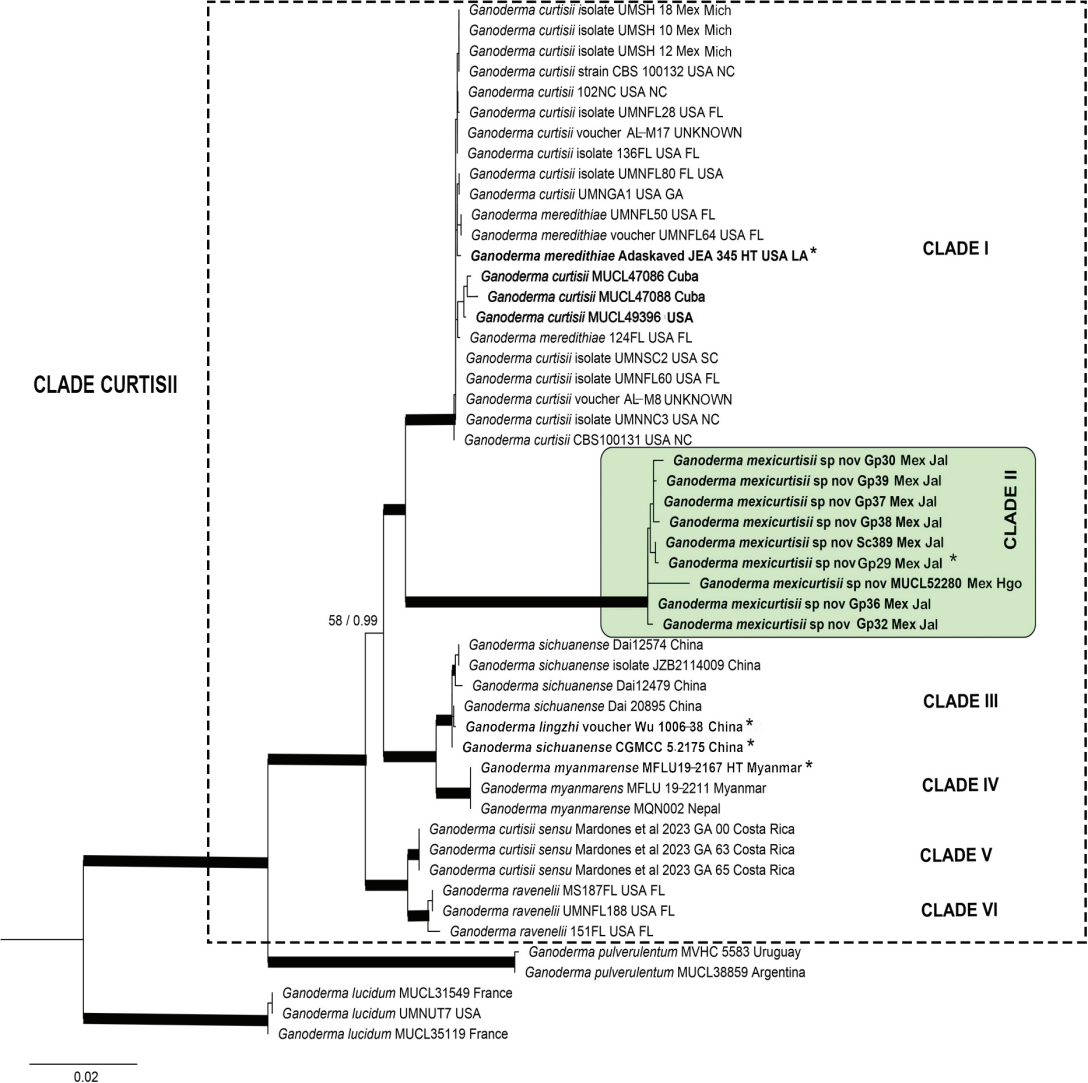
Phylogeny of the *Ganodermacurtisii* lineage. Inferred from concatenated ITS, *rpb1*, *rpb2*, and *tef1* (3581 bp) DNA sequences, using maximum likelihood. Thickened branches represent bootstrap support values greater than 75% and Bayesian posterior probabilities greater than 0.95. Sequences in bold were obtained in this work, *represent type specimens.

In the BI analyses, both Bayesian runs converged to stable likelihood values after 1160000 generations. Therefore, 8840 stationary trees from each analysis were used to compute a 50% majority rule consensus tree and to calculate PP. In the ML searches, the combined dataset alignment had 762 distinct patterns with a proportion of gaps and undetermined characters of 44.35%. The phylogenetic trees obtained from BI and ML inferences using the concatenated data sets showed overall the same topology and the ML tree is presented in Fig. 1.

The phylogenetic inferences resulted in six terminal clades within the “curtisii clade” (Fig. 1, BS 100 / PP 1). Clade I (BS 87 / PP 0.99) corresponds to *G.curtisii* s.s. and is composed of collections from southeastern USA, Cuba, and Mexico, including sequences from the holotype culture of *G.meredithiae*. Clade II (BS 100 / PP 1), *Ganoderma* sp., is sister to Clade I, and comprises only specimens from Mexico. Clade III (BS 98 / PP 1) corresponds to *G.sichuanense* (= *G.lingzhi*) and is the sister to clade IV (BS 100 / PP 1), which corresponds to *G.myanmarense* Karunarathna, Mortimer & Luangharn. Clade V (BS 75 / PP 0.91) corresponds to *G.curtisii* sensu Mardones et al. (2023) and includes three collections from Costa Rica. Clade VI (BS 82 / PP 0.99) corresponds to *G.ravenelii* and includes specimens from the Southeastern USA.

### ﻿Morphology

The specimens forming clades I and II are morphologically very similar. They are characterized by mainly lateral, rarely centrally stipitate basidiomes, with light yellow to red pileus surface, a light, duplex context, with none to several (up to 4) dense, pale to brown stripes or continuous lines of resinous deposits in the context, extending from the pileus margin toward the stipe. Their cuticular cells are mainly cylindrical to clavate, apically rounded, and the basidiospores are oblong to ellipsoid, with subfree pillars. They have subtle differences in morphology and reaction in Melzer’s reagent of the cuticular cells, which are entire or rarely with one branch and amyloid in specimens of Clade I, while they are entire, branched or vesiculated and slightly to rarely strongly amyloid, to almost black in mass, in specimens of Clade II.

### ﻿Taxonomic conclusions

Specimens forming clades I and II are very similar in their gross morphology, with only elusive differences in the cuticular cell morphology, which, with careful analysis, could allow to distinguish two morphotypes that in turn could be considered as two morphospecies. Furthermore, each morphotype corresponds concordantly to a distinct clade or phylogenetic taxon. We therefore conclude that the specimens from Mexico forming clade II represent a species on its own, described here as *G.mexicurtisii*. The differences of the *G.mexicurtisii* sequences with those of the clade of *G.curtisii* s.s. were: ITS a single nucleotide polymorphism (A–G in ITS2), eight nucleotide polymorphisms (C–A, C–G, C–T, G–A, G–T, and T–C) in exons of *tef1*, six (A–G, C–T, G–A, G–T, and T–C) in exons of *rpb2*.

### ﻿Taxonomy

#### ﻿ Ganoderma mexicurtisii

Taxon classificationAnimaliaPolyporalesPolyporaceae

Cabarroi-Hernández, Decock, Amalfi, M.Torres & Guzm.-Dáv. sp. nov.

CD77041D-A10A-5D03-8D65-CDFB6FD55A12

859381

Fig. 2

##### Diagnosis.

*Ganodermamexicurtisii* is similar to *G.curtisii* but distinguished by a generally paler yellowish pileus and clavate cuticular cells, apically branched or with protuberances and spheroid diverticula, slightly to strongly amyloid.

##### Type.

MEXICO, JALISCO: • Municipality of Mixtlán, taken from the exhibition in the Mushroom Fair in Mixtlán, oak-pine forest, on roots, 18 Jul 2021, L.Guzmán-Dávalos 12340 (holotype: IBUG-15648!, isotypes: BR!, NY!), sequences ITS rDNA, *rpb2*, and *tef1* (Gp29).

##### Description.

Basidiome stipitate, solitary, sometimes scattered, rarely two from the same base, spongy when fresh, light in weight. Pileus dimidiate, conchate in pole view, rarely circular, convex or applanate in section, projecting 4–9 cm, 5–15 cm wide; pileal surface laccate to dull, smooth, rugose or with concentric deep sulcations, pale yellow (4A3, 4A6), deep yellow (4A8), yellowish–orange (5B8), yellow–brown (5C8) to brown–violet (11F8), generally darker near the union with the stipe, peeling off very easily, exposing the context; margin regular to slightly lobulated, sometimes incurved, white to pale yellow (4A3) to light brown (7E8). Stipe mostly mesopodal, occasionally pleuropodal, mainly orthopleuropodal, occasionally plagiopleuropodal, or rarely dorsally lateral, cylindrical, straight or bended to tortuous, 2.5–27 × 1–5 cm, uniform, slightly swollen or attenuate at the base, laccate, brown–violet (11F8), golden–yellow (4A4, 5B8), concolor with the pileus to pale yellow (3A3). Pore surface pale yellow (2A3) when fresh, pale yellow (3A3) to golden–yellow (4A4, 5B8) on drying, staining brown (6D8) when bruised; pores angular, occasionally round, 2–4 (–5) per mm; dissepiments conspicuous. Context duplex, with a spongy consistency, slightly fibrous texture, 0.7–2(–4) cm thick, pale to light orange (5A3–5A4) next to the crust, and brown–sienna (6D7) to rarely yellow–brown (6F8) above the tubes, with few to several (up to 4) longitudinal, pale to brown, resinous bands, azonate. Tubes 3–10 mm deep, without stratification, rarely stratified, brown–violet (8E4).

Hyphal system dimitic; generative hyphae 3–3.5 μm diam., septate, with clamp connections, thin–walled, hyaline to yellowish, difficult to observe; skeleto–binding hyphae with an arboriform pattern, pale golden–yellow, tortuous, sometimes with some protuberances, golden–yellow, thick–walled, composed of a basal stalk arising from a clamp, with apical processes, the branches gradually tapering from 3.2–4 μm wide at primary processes to 2.4–3.2 μm wide at thin–walled apices in an area above the tubes, and from 4–6 μm wide at primary processes to 3–4 μm wide at the thin–walled apices with some moniliform hyphae (7–11 µm diam.) below the crust. Pileipellis a crustohymeniderm; cuticular cells mostly clavate, lumen widely open, in the lighter zone of the pileus, thin–walled, 0–3 protuberances, with several or non spheroid diverticula, slightly amyloid to almost black in mass, and in the darker zone of the pileus clavate, with no or ≤ 4 irregular long branches, rarely the clavate form lost, thick–walled, strongly amyloid, 43–96 × 7.2–16.5 µm. Basidia pyriform, with four sterigmata, ~ 20 × 7.2 μm. Basidiospores oblong to ellipsoid, with an apical conical umbo often collapsed, then appearing truncate, double–walled, the inner wall thick, pale yellowish, with thick, subfree pillars, (9.2–) 10.5–12.8 (–13.5) × 5.5–8.0 µm, Q = 1.44–1.88. Chlamydospores not observed.

##### Habitat and distribution.

Solitary to scattered, growing on soil (roots of trees, mainly *Quercus*), in oak-pine forest and a relict mixed forest of Fagusgrandifoliavar.mexicana and *Pinus*. Known from Mexico (Hidalgo, Jalisco, Morelos, State of Mexico).

##### Etymology.

Prefix “mexi” refers to Mexico and “curtisii” refers to its resemblance to *Ganoderma curtisii*.

##### Specimens examined

**(all as paratypes of *Ganodermamexicurtisii*).** MEXICO, HIDALGO: • Municipality of Tenango de Doria, Temapa, 20°37'47"N, 98°12'49"W, 20 Nov 1969, J.Gimate 152–A (ENCB); • Municipality of Zacualtipan, 20°37'47"N, 98°36'58"W, 2000 m a.s.l., relictual mixed Fagusgrandifoliavar.mexicana and *Pinus* forest, emerging from leaf-litter, 22 Jul 2009, C.Decock MX–09–20 (living strain MUCL 52280 = CBS 152593). JALISCO • Municipality of Bolaños, Crucero Miguelón, 21°55'17.7"N, 103°52'13.9"W, pine-oak forest, 30 Aug 2024, M.Cabarroi 20 (IBUG); • Municipality of Colotlán, 16.5 km west of Colotlán to Carrizal, 22°5'1.23"N, 103°12'24.8"W, 2 Aug 2004, M.G.Torres-Torres 541 (IBUG); • Municipality of Cuautitlán de García Barragán, brecha Las Joyas-Manantlán, 19°33'10"N, 103°46'19.99"W, 23 Sep 1983, A.G.Valenzuela s.n. (IBUG); • Municipality of Cuautla, Tierras Blancas, 20°15'39"N, 103°30'20.99"W, 2200 m a.s.l., pine-oak forest, 16 Jul 2017, L.Guzmán-Dávalos 11569 (IBUG), sequence Gp30; • 29 Jul 2018, L.Guzmán-Dávalos 11735 (IBUG), sequence Gp38; • Municipality of Cuquío, Las Cruces, 21°1'25"N, 102°53'20"W, 12 Oct 1980, J.Mejía-Jimenez s.n. (IBUG); • Municipality of Etzatlán, Etzatlán-Mesa Colorada road, near El Amparo, 20°42'3"N, 103°55'1.99"W, 1600–1800 m a.s.l., oak-pine forest, 4 Sep 1997, L.Guzmán-Dávalos 6742 (IBUG); • Municipality of Mascota, 800 m after La Campana, km 83.5 Guadalajara-Mascota road, 20°42'3"N, 103°55'1.99"W, pine-oak forest, 17 Aug 1998, L.Guzmán-Dávalos 7447; • 16 Jul 2022, L.Guzmán-Dávalos 12404 (IBUG); • Municipality of Mazamitla, 5 km west of Mazamitla, Los Cazos, 19°54'7"N, 102°57'57.99"W, 15 Feb 1994, H.Orozco 5 (IBUG); • 5 km from La Manzanilla de La Paz to Mazamitla, 19°57'14"N, 102°49'59"W, pine-oak forest, 6 Oct 1984, L.Guzmán-Dávalos 1723 (IBUG); • Municipality of Mezquitic, La Cebolleta, pine-oak forest, on *Quercus* sp., 15 Aug 1997, L.Villaseñor-Ibarra 282 (IBUG); • Municipality of Mixtlán, Los Perones, 20°26'45.2"N, 104°21'53.99"W, 28 Jul 2018, M.X.Haro-Luna 394 (IBUG), sequence Gp37; • Mesa Colorada, 20°28'43.31"N, 104°22'29.43"W, oak-pine forest, 27 Jul 2018, O.Castro-Jauregui 365 (IBUG), sequence Gp36; • 19 Jul 2023, on stump of *Pinus* sp., M.Cabarroi 12 (IBUG), sequence Sc389; • Municipality of Tecolotlán, Sierra de Quila, km 17.5 Tecolotlán-Quila road, 20°18'14"N, 103°57'3.99"W, M.L.Fierros 494–A (IBUG); • Municipality of Tequila, Tequila volcano, km 8 of the road to the microwave station, 20°47'31"N, 102°8'45.99"W, oak-pine forest, 30 Jul 1986, J.A.Pérez de la Rosa s.n. (IBUG); • km 12 of the road to the microwave station, oak-pine forest, 28 Jul 2017, L.Guzmán-Dávalos 11586 (IBUG), sequence Gp31; • 18 Aug 2018, L.Guzmán-Dávalos 11754 (IBUG), sequence Gp39; • Municipality of Puerto Vallarta, km 31–32 of the Mascota-Puerto Vallarta road, 20°43'21.47"N, 104°52'30.18"W, 10 Sep 2005, P.G.Castañeda 5 (IBUG); • Municipality of Zapopan, Huaxtla, on the side of the road to Cuisillos, on *Quercus* root, Jun 2024, S.Mata s.n. (IBUG); • Bosque La Primavera, 3 km SE of La Primavera town, 20°39'11.65"N, 103°32'24.50"W, oak-pine forest, 20 Oct 1983, L.Guzmán-Dávalos 1277 (IBUG); • aprox. 8 km from Mariano Otero Avenue, 20°36'16.54"N, 103°32'26.60"W, oak-pine forest, 20 Jul 2004, M.G.Torres-Torres 526 (IBUG); • Bosque La Primavera, entrada por ejido Emiliano Zapata, 20°41'57.75"N, 103°37'56.92"W, 14 Jul 2013, oak forest, M.Hererra 1564 (IBUG); • Bosque La Primavera, 20°41'53.4"N, 103°34'34.7"W, oak-pine forest, 4 Aug 2023, R.Betancourt 89; • Bosque del Centinela, corner of Avenue Bosques del Centinela with the street Bosque de Nayarit, 20°45'48.84"N, 103°22'30.25"W, induced oak-pine forest with eucalyptus, casuarina, and castor oil plant, 7 Jul 2018, O.Castro-Jauregui 146 (IBUG), sequence Gp32. MORELOS • Municipality of Tepoztlán, 5 km west of Tepoztlán, near the highway to Cuautla, 18°59'45.98"N, 99°9'40.85"W, 3 Sep 1967, M.Frias Neve 18 (ENCB). STATE OF MEXICO • Municipality of Texcoco, Tetzcotzinco Archaeological Zone, on the way to Conjunto Norte, 19°29'50.55"N, 98°49'9.85"W, on *Quercus* root, 14 Nov 2024, M.Cabarroi 21 (IBUG).

**Figure 2.**
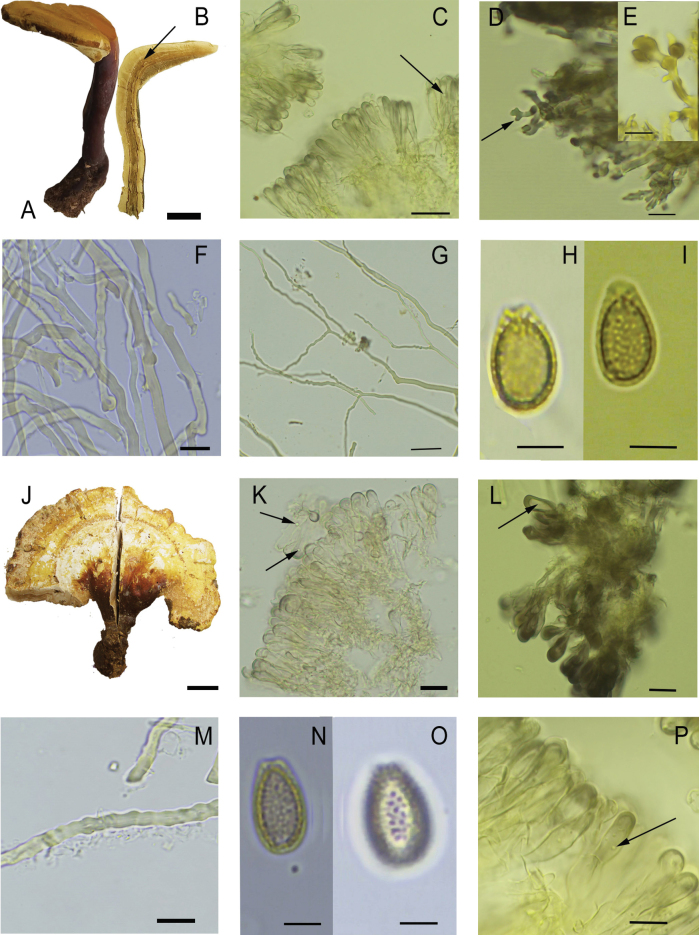
*Ganodermamexicurtisii*. **A–I** L.Guzmán-Dávalos 12340 (IBUG, holotype), **A** orthopleuropodal basidiome, **B** duplex context with longitudinal resinous bands (arrow), **C** cuticular cells from the paler pileal surface with vesicles (arrow), **D–E** cuticular cells from the darker pileal surface with some apical diverticles (arrow), **F** moniliform hyphae below the crust, **G** skeleto–binding hyphae with an arboriform pattern, **H–I** basidiospore with subfree pillars. **J–O** M.Cabarroi 12, **J** plagiopleuropodal basidiome, **K** cuticular cells from the paler pileal surface with vesicles (arrows), **L** clavate cuticular cells from the daker pileal zone, with lumen widely open (arrow), **M** moniliform hyphae below the crust, **N–O** basidiospore with subfree pillars. **P** R. Betancourt 89, cuticular cells from the paler pileal surface with vesicles (arrow). Scale bars: 2 cm (**A, B**); 20 µm (**C, D**); 10 µm (**E, F, G, K, L, P**); 5 µm (**H–I, M–O**); 1 cm (**J**). Photo **A** Laura Guzmán-Dávalos, **B–P** Milay Cabarroi-Hernández.

##### Specimens of *Ganodermacurtisii* examined.

**Figure 3.**
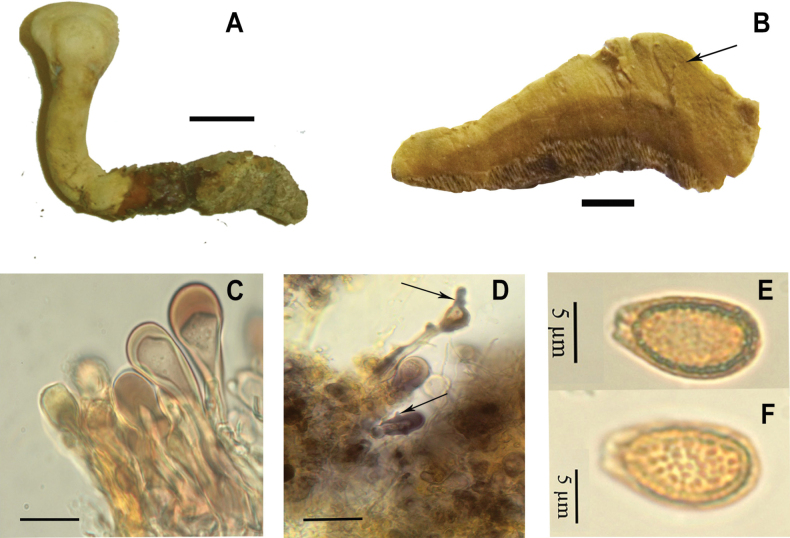
*Ganodermacurtisii*, C.Decock CU–05–224 (MUCL 47086). **A** Ortho- to plagiopleuropodal basidiome, **B** duplex context with a resinous band (arrow), **C** clavate cuticular cells, with lumen widely open, **D** cuticular cells with occasionally apical branches (arrow) and protuberances (arrow), **E–F** basidiospore with subfree pillars. Scale bars: 4 cm (**A**); 1 cm (**B**); 10 µm (**C, D**). Photos **A–C, E, F** Milay Cabarroi-Hernández, **D** Cony Decock.

CUBA, PINAR DEL RÍO • Guanahacabibes península, Municipio Sandino, finca del guarda forestal, 1 km from Cayuco, oak forest, on *Quercuscubana* stump, 28 Sep 2005, C.Decock CU–05–224 (MUCL 47086, living strain MUCL 47086) (Fig. 3); • C.Decock CU–05–225 (MUCL 47087); • C.Decock CU–05–227 (MUCL 47088, living strain MUCL 47088). USA, NORTH CAROLINA • Chapel Hill, Nov 1923, Coker s.n. (RLS.63.K.59 & K.58), isoneotypus at BR!, fragm.); • Forsyth, Winstom-Salem, in yard, 30 Sep 1934, Schallert s.n. (F); GEORGIA • Thomas, Thomasville, 19 Jul 1947, Field s.n. (F); LOUISIANA • Calcasieu, Lake Prien, on the ground in pine woods, 30 Oct 1984, Drouet s.n. (F).

##### Specimens of *Ganodermaravenelii* examined.

USA, SOUTH CAROLINA • Aiken, on the ground, s. data, Ravenel 2936 (K, holotype!); FLORIDA • Columbia, Camp O’Lena State Park, s. data, Dybas s.n. (F).

## ﻿Discussion

We found six terminal clades in the “curtisii clade” sensu Loyd et al. (2018a), with Clade I corresponding to *G.curtisii* s.s. or sensu Loyd et al. (2018b). *Ganodermacurtisii* has been widely reported in Mexico, with a great intraspecific morphological variability (e.g., Guzmán 1977; Torres-Torres and Guzmán-Dávalos 2005; Torres-Torres et al. 2015; López-Peña et al. 2016; Capello-García et al. 2023; Mendoza-Churape et al. 2024). Excluding Mendoza-Churape et al. (2024), most of the works that have recorded *G.curtisii* from Mexico have been based on morphological data. Considering the high variability reported and the results of this study, we suggest that many of the specimens previously identified as *G.curtisii* sensu Mexican authors correspond to the new species, *G.mexicurtisii*. This species can be considered cryptic, as it cannot be morphologically distinguished from *G.curtisii* s.s., but instead forms a distinct phylogenetic lineage based on DNA sequences (Peintner et al. 2019).

*Ganodermamexicurtisii* is characterized by stipitate, meso- to pleuropodal basidiome, mainly orthopleuropodal (lateral and vertical), occasionally plagiopleuropodal (lateral and horizontal), with generally a pale–yellow pileus, sometimes brown–violet, and a duplex context with darker resinous bands, which may fade towards the margin. The specimens of *G.mexicurtisii* examined show variability in their cuticular cells, from slightly amyloid with many branches and diverticula or spheroid vesicles to strongly amyloid, entire, or with up to four branches and without diverticula or vesicles. This range of variation is observed even within the same specimen. Therefore, cuticular cell morphology and reaction in Melzer’s reagent should not be considered as a strictly reliable differentiating character between *G.curtisii* and *G.mexicurtisii*.

Although forming two well-distinct clades, as mentioned *G.mexicurtisii* is morphologically very similar to *G.curtisii*, with only slight differences of uncertain interpretation. *Ganodermacurtisii* is characterized by a darker pileus color, ranging from yellowish–orange to reddish–brown, often with purple hues, while *G.mexicurtisii* is characterized by a paler yellow to yellowish–orange, rarely violet–brown pileus. It was described with mostly entire, more rarely 0–2 lateral or apical branched cuticular cells (Steyaert 1980; Torres-Torres and Guzmán-Dávalos 2008). Haddow (1931) noted cuticular cells “more or less in disorderly array in light coloured part”, which we were able to corroborate in a fragment of the isoneotype available at BR. However, taking into account the synonymy of *G.meredithiae* with *G.curtisii* (Loyd et al. 2018a, Fig. 1), we should consider that the cuticular cells of *G.curtisii* are more variable than previously described. *Ganodermameredithiae* was described with “lobed or branched” cuticular cells (Adaskaveg and Gilbertson 1988).

The duplex context and resinous bands are also present in *G.curtisii*, as noted by Steyaert (1980), who described the context of *G.curtisii* as “pale ochraceous buff to ochraceous, tawny near tube layer”. We observed in both *G.curtisii* and *G.mexicurtisii* a light context, generally pale orange next to the crust and brown sienna near the tube layer. The illustrations of Mendoza-Churape et al. (2024) as *G.curtisii* from Mexico are different from the concept of this species because they showed basidiomata with no duplex and distinctly browner context. Furthermore, they showed a gregarious even imbricated basidiomata, different from the growth that has been observed in *G.curtisii*. The ITS region of these Mexican collections differed by a nucleotide position from the ITS of *G.curtisii* from the USA. Unfortunately, the *tef1* and *rpb2* sequences (Mendoza-Churape et al. 2024) were not available to us, impeding comparison. The Cuban collections of *G.curtisii* (Cabarroi-Hernández et al. 2023) formed a slightly divergent subclade within the *G.curtisii* clade (Fig. 1).

The distribution range of *G.curtisii* and *G.mexicurtisii* could be another feature to take into account. *Ganodermacurtisii* is found in the southeastern United States or, in ecological terms, in the “warm continental” to “subtropical” divisions (Perry et al. 2022). This corresponds, roughly, to the southeastern plains and the southeastern coastal plain, in oak and pine forests (or the Gulf Coast region from East Texas to Georgia). Its northern and western limits of distribution in the United States are uncertain. Records from west of the Great Plains and southern Canada / northeastern USA should be critically revised. Southerly, *G.curtisii* has been confirmed in the westernmost peninsula of Cuba on *Quercuscubana*, in oak forest (Cabarroi-Hernández et al. 2023). In Mexico, it has been cited from the State of Michoacán, western Mexico, in plantations of *Perseaamericana* (Mendoza-Churape et al. 2024). The westernmost peninsula of Cuba and the State of Michoacán (Mexico) represent its southernmost distribution to date.

*Ganodermameredithiae* was originally distinguished from *G.curtisii* based on a presumed host specificity for pines. *Ganodermameredithiae* was also differentiated by more diverticulated cuticular cells and a faster *in vitro* mycelial growth rate (Adaskaveg and Gilbertson 1988). However, Loyd et al. (2019) and our current phylogenetic inferences found no phylogenetic support for the recognition of *G.meredithiae* as a distinct species and both names are here considered synonymous. This fact greatly increased the plasticity of the basidiomata of *G.curtisii*.

**Figure 4.**
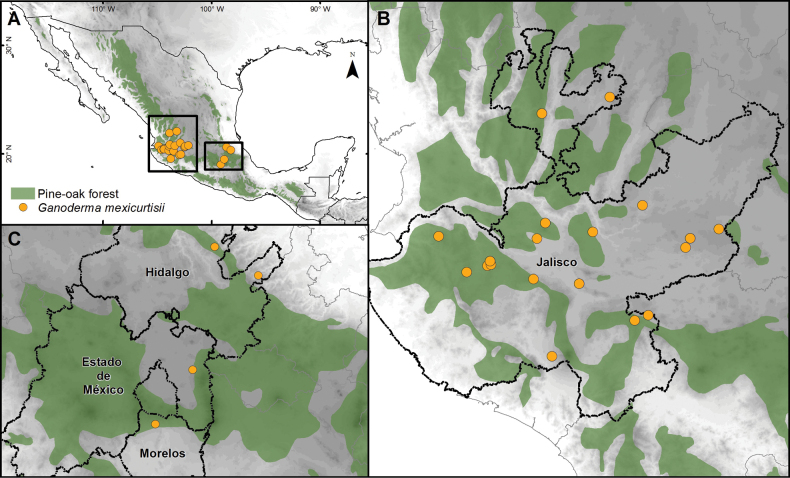
Geographic distribution of *Ganodermamexicurtisii* in Mexico. **A** Distribution in the country, **B** in Jalisco, **C** in Estado de México, Hidalgo, and Morelos.

To date, *G.mexicurtisii* is only known from Mexico, mainly inhabiting *Quercus*-*Pinus* forests, and was also found in a relict mixed forest of Fagusgrandifoliavar.mexicana and *Pinus* (C. Decock MX–09–20). It is widespread in central Mexico, found in Estado de México, Hidalgo, and Morelos, and widely abundant in the western state of Jalisco (Fig. 4). Our specimens, for which the host was identified, were found growing on both *Quercus* spp. and *Pinus* spp. Similarly, the *G.curtisii* clade (Fig. 1) of this study brought together specimens growing on both *Quercus* and *Pinus* spp. (Loyd et al. 2019), as well as on *Perseaamericana* (Mendoza-Churape et al. 2024).

*Ganodermamexicurtisii* is, along with *G.curtisii* and *G.ravenelii*, the third named species of the *G.curtisii* lineage present in the New World. Specimens tentatively identified as *G.curtisii* were also reported from the highlands (1800 and 2600 m a.s.l.) of Costa Rica in oak and pine forests, mostly growing on oak, more rarely on pines (Mardones et al. 2023). In our phylogenetic inferences, these Costa Rican specimens formed an independent clade, sister to the *G.ravenelii* clade (Fig. 1), thus they represent an additional fourth New World species. The American species of the “curtisii clade” mainly inhabit oak and pine forest ecosystems, and they could be searched for within the whole range of oak or oak-pine forests in Mesoamerica and southward down to Panama or Colombia. In Mesoamerica and Colombia, oak or oak-pine forests are found mainly in mountainous areas above 1000 m elevation (Nixon 2006). Currently, Costa Rica represents its southernmost distribution limit. In that sense, reports of *G.curtisii* from Brazil (Torres-Torres et al. 2012) likely represent another species. However, no DNA data is available so far to infer the taxonomic placement and affinities of these Brazilian collections.

Comparatively, only two species of the “curtisii clade” are known to date from East Asia, viz. *G.myanmarense* and *G.sichuanense*, known from China, Myanmar, and Thailand (Cao et al. 2012; Luangharn et al. 2021; Du et al. 2023). Both species also inhabit mainly warm temperate to subtropical fagaceous forests, growing on *Quercus* or phylogenetically related taxa such as *Castanopsis* and *Cyclobalanopsis* (Du et al. 2023). Although Lloyd (1915) reported *G.curtisii* from tropical Africa, no African specimens are known to date within the “curtisii clade”, so we consider them absent from the African continent.

Species of the “curtisii clade” have a disjunct distribution between, on one side, the southeastern United States, Mesoamerica, and Cuba, and, on the other, subtropical to tropical eastern Asia. This type of distribution is a well-known phytogeographical pattern for Northern Hemisphere vegetation (e.g., Donoghue and Smith 2004). It remains to be seen whether the distribution pattern of the “curtisii clade” can be understood in the same context. To date, biogeographic patterns have not been extensively studied in *Fungi* (Mueller et al. 2001). It should nevertheless be noted that American species of the “curtisii lineage” belong to two distant clades (I–II and V–VI). *Ganodermacurtisii* and *G.mexicurtisii* are sister clades, as are *G.ravenelii* and *Ganoderma* sp. However, the V–VI clade is basal to the “curtisii lineage” as a whole, including the Asian species *G.myanmarense* and *G.sichuanense*, suggesting a complex evolutionary biogeography.

It should be highlighted the potentially medicinal importance of *G.mexicurtisii*. Welti et al. (2015) and Islas-Santillán et al. (2018) reported bioactive compounds in extracts of *G.curtisii* from Mexico, which most likely correspond to *G.mexicurtisii*. Welti et al. (2015) analyzed the strain MUCL 52280, which was nested in the *G.mexicurtisii* clade in this work. Therefore, the four lucidenic acids, as well as two other partially identified compounds, which the authors referred to as extracted from *G.curtisii*, could belong to *G.mexicurtisii*. Similarly, Huerta Aguilar et al. (2016) found antioxidant properties in hydroalcoholic and ethanolic extracts of *G.curtisii* from the state of Michoacán, Mexico, which could also refer to *G.mexicurtisii*. Therefore, *G.mexicurtisii* requires further chemical studies to corroborate the presence of compounds with medicinal properties. Given the morphological overlap between these species, *G.curtisii* and *G.mexicurtisii*, and the likelihood of misidentification, it is essential to expand and deepen chemical and pharmacological studies on *G.mexicurtisii*. This will help confirm its medicinal potential and prevent the misattribution of bioactive properties between these closely related taxa.

## Supplementary Material

XML Treatment for Ganoderma mexicurtisii

## ﻿Additional information

### Author ORCIDs

Milay Cabarroi-Hernández https://orcid.org/0000-0002-5632-4456

Cony Decock https://orcid.org/0000-0002-1908-385X

Laura Guzmán-Dávalos https://orcid.org/0000-0002-4390-3678

Gerardo Lucio Robledo https://orcid.org/0000-0002-3840-1104

Virginia Ramírez-Cruz https://orcid.org/0000-0002-5197-1304

Mario Amalfi https://orcid.org/0000-0002-1792-7828

### Data availability

Sequence data ara available in GenBank (see Table 1) and alignment in Figshare 10.6084/m9.figshare. 29218322.
